# The Construction and Validation of Child, Adolescent and Parental Decision Aids for Considering Methylphenidate Drug Holidays in ADHD

**DOI:** 10.3390/pharmacy6040122

**Published:** 2018-11-24

**Authors:** Kinda Ibrahim, Gina Randolph, Olivia Doran, Parastou Donyai

**Affiliations:** 1 NIHR CLAHRC Wessex, University Hospital Southampton NHS Foundation Trust, Faculty of Medicine, University of Southampton, Southampton SOA6 6YD, UK; 2 Reading School of Pharmacy, University of Reading, P.O. Box 226, Whiteknights, Reading RG6 6AP, UK; gina_randolph@hotmail.co.uk (G.R.); obdoran@live.co.uk (O.D.); p.donyai@reading.ac.uk (P.D.)

**Keywords:** ADHD, drug holiday, decision aid, children and adolescents

## Abstract

Guidelines recommend encouraging young people with attention deficit hyperactivity disorder (ADHD) who are taking medication long-term, to discuss their preferences for stopping or changing their treatment, including a discussion about ‘drug holidays’, with their doctor. Yet, to date, no written information has been available to empower children and adolescents with ADHD and their parents to make informed decisions about drug holidays. The aim of this study was to design and develop a suite of decision aids to help families decide if they want to take a drug holiday from methylphenidate. The material was designed with reference to the literature and in consultation with a secondary-care specialist, and validated with two panels composed of specialists and parents using content validity questionnaires and interviews; before being finished and branded by a design service. Three decision aids were produced, with parental and adolescent versions composed of a booklet and a pull-out form for self-completion, and the child version being a booklet for reading and self-completion. Existing research calls for suitable written materials to feasibly increase the uptake of practitioner-initiated planned drug holidays from methylphenidate. We envisage these materials will open up the space to discuss drug holidays in ADHD during annual reviews, in line with UK government guidelines.

## 1. Introduction

Medicines can produce unwanted or unanticipated adverse reactions, also known as side-effects. Adverse drug reactions are graded according to their intensity and most manufacturers provide a frequency estimate for side-effects from very common (≥10%) to common (1–<10%), uncommon (0.1–<1%), rare (0.01–<0.1%), very rare (<0.01%), or not known (cannot be estimated from available data). Another aspect relating to side-effects is their duration, for example whether they are transient or long-lasting. No drug is without side-effects, therefore medicine use is often linked with the term ‘benefit–risk ratio’ both at a general level, in terms of approval for use by regulatory agencies, and at an individual level, in terms of whether prescribers and patients decide to use them [1]. In considering the benefits of medication, four main aspects include the seriousness, the time course and incidence of the disease against the extent of improvement, the reduction in time, and the incidence of improvement of the disease with the medication [1]. The risks can be considered against the intensity, frequency, or duration of side-effects.

One particular area of interest is the drug methylphenidate for the treatment of child and adolescent attention deficit hyperactivity disorder (ADHD), where parents’ dilemmas regarding treatment and side-effects was captured by Hansen and Hansen [2] with the phrase ‘caught in a balancing act’. ADHD is a behavioral disorder that often becomes obvious in early childhood because of the underlying problems with poor attention, hyperactivity, and impulsivity [3]. Methylphenidate is a centrally acting sympathomimetic, thought to inhibit dopamine reuptake in the striatum, without triggering the release of dopamine; resulting in functional improvements in ADHD [4]. Many studies have documented the benefits of methylphenidate in improving behavioral symptoms [2,5,6]. Worldwide, studies have shown that ADHD medications (both stimulants and non-stimulants) are effective in reducing ADHD symptoms and enhancing academic functioning in children receiving treatment [7,8,9]. Yet, methylphenidate results in a number of common and very common side-effects including ‘anorexia, decreased appetite, moderately reduced weight and height gain during prolonged use in children’ [4] and has been associated with disturbance of sleep functioning as well as a reduction in childhood growth [10]. Accordingly, drug manufacturers recommend that extended use (>12 months) should be accompanied by periodic re-evaluation of the drug with trial periods off medication [4]. This is also recommended by guidelines internationally [11] and supported in the United Kingdom (UK) by the National Institute for Health and Care Excellence (NICE) [12]. Trial periods off medication are also known as ‘drug holidays’; ‘a deliberate interruption of pharmacotherapy for a defined period of time and for a specific clinical purpose’ [13]. Methylphenidate’s risk profile thus warrants close scrutiny of the practice of drug holidays in ADHD.

Using grounded theory to specifically examine the views and experiences of planned drug holidays from methylphenidate, Ibrahim, Vogt and Donyai [14] reported a core category, ‘caught in the eye of the storm’, whereby parents are in effect spellbound to continue medication-giving due to the experienced benefits of methylphenidate, despite the side-effects. Interestingly, later in their treatment, adolescents take a lead in stopping their medication altogether even though some might not be able to manage their condition without medication. The health professionals reported a lack of engagement with methylphenidate drug holidays [14]. This was despite the fact that locally, under shared-care arrangements, prescribers were asked to plan two-week drug holidays with patients and their families after two years of treatment, to test their continuing need. Older research has also shown that only 30% and 60% of practitioners in the United States (US) and the UK, respectively, would consider discussing drug holidays annually with families of children with ADHD [15,16]. This is despite evidence that longer breaks from methylphenidate could help with child growth, with shorter breaks reducing insomnia and improving appetite [17]. In the UK, NICE guidelines that recommend “A healthcare professional with training and expertise in managing ADHD should review ADHD medication at least once a year and discuss with the person with ADHD (and their families and carers as appropriate) whether medication should be continued” [12].

A psychological framework for explaining human behavior was used by Ibrahim and Donyai to analyze the transcripts of interviews with health professionals, to identify the specific barriers to engagement with planned drug holidays from methylphenidate [18]. The COM-B framework stipulates that three components, capability, opportunity, and motivation, interact to explain a particular behavior, and has been proposed as a basis for the selection and design of behavior-change interventions [19]. The analysis by Ibrahim and Donyai identified ‘opportunity’ (lack of written information about drug holidays) as a key barrier to engagement with planned drug holidays from methylphenidate. ‘Enablement’ and ‘engagement’ interventions were identified as key activities targeting the barriers identified, to feasibly increase the uptake of practitioner-initiated planned drug holidays from methylphenidate. According to NICE [12], people with ADHD should be encouraged to discuss any preferences to stop or change medication and to be involved in any decisions about stopping treatments. Yet, to date, no written information has been available to aid shared-decision making with children and adolescents with ADHD and their parents. This was confirmed through a strategic search of the database PubMed on 5 November 2018. Thus the aim of this study was to design and develop a decision aid to help families make informed decisions about whether or not to implement methylphenidate drug holidays.

## 2. Materials and Methods

The study employed a sequential approach whereby existing guidelines and research findings informed the development of, firstly, a paper-based decision aid intended for use by parents, the scientific content of which was developed by working with an expert, and then validated with an additional group of secondary-care practitioners (n = 4). The decision aid was then modified to produce additional adolescent and child-friendly versions before all three versions of the decision aid were formatted and branded with the help of a university design service. The visual presentation and clarity of these formatted versions were then validated with the help of a panel with mixed expertise (n = 9), before being modified again to produce the final versions. According to researchers, the number of people within a panel judging content validity should be between 3 and 10 with >10 deemed unnecessary [20]. This was the guiding principle for recruitment of the two panels (n = 4 and n = 9) who reviewed these decision aids. The study was approved by the University of Reading Research Ethics Committee (reference UREC 12/18 and amendment email 16/1).

### 2.1. Development and Validation of the Parental Decision Aid for Methylphenidate Drug Holidays

The initial content of the parental decision aid (version 1; v1) was developed by authors KI and PD using the published literature and adhering to the criteria established by the International Patients Decision Aids Standards (IPDAS) [21], with further input from a Child and Adolescent Mental Health (CAMH) consultant. The content was designed to contain information that was relevant to decisions about taking a drug holiday, while aiming to be simple and easy to understand. It consisted of four main sections: (1) Frequently asked questions, to provide comprehensive information about planned drug holidays from methylphenidate; (2) Compare options, to explain the risks and benefits of considering a planned break from the medication; (3) Your feelings, to allow the individual to test their subjective feelings and weigh up their receptiveness to the idea of a drug holiday; and (4) Your decision, to document a final decision about whether or not to introduce a planned drug holiday from ADHD medication. The format mimicked an existing web and paper-based decision aid developed for use by parents to consider the need for medication in children with newly-diagnosed ADHD [22].

It was deemed appropriate to determine the content validity index (CVI) of the decision aid to ascertain its content validity (CV). This is because the CVI measures the extent of agreement between experts about each item of a newly-developed tool, such as the current decision aid (hereon referred to as the item CVI; I-CVI), and helps improve each item where needed [23]. The decision aid was thus divided into 20 items so that each item represented a single statement or clear grouping of statements within the text. A panel of four CAMH practitioners working within one community and mental health hospital trust was recruited, through an existing CAMH contact who emailed invitation letters (to circa 30 practitioners), to take part in the CV exercise. Each panel member was asked to read each of the items on the parental decision aid and judge the relevance of the material on a four-point response scale, providing additional comments where appropriate; with 4 being used to indicate item being very relevant, 3, mostly relevant but needs some revision, 2, mostly not relevant and needs more revision, and 1, not relevant.

The I-CVI was determined by calculating the proportion of panel members who gave a rating of 3 or 4 (relevant or mostly relevant) for each item of the decision aid. An I-CVI of 1 (four ratings of 3 or 4 from four ratings) was judged as the acceptable value for responses about each item, meaning all four panel members had to judge the item to be relevant or mostly relevant. This acceptable value was based on Lynn’s criteria [20] and any item not receiving the acceptable value was either removed or modified to produce a second version (v2). In addition to this, the validity of the entire document was also determined by calculating the proportion of all items that achieved a rating of 3 or 4 by all the content experts, which is normally acceptable at >0.80 [24].

### 2.2. Development of the Child- and Adolescent-Friendly Versions of the Decision Aid

Based on the parental decision aid (v2) for methylphenidate drug holidays, two additional forms were developed targeting younger children (aged 9–12 years) and adolescents (aged 13–17 years) with ADHD, with reference to guidance for designing child-friendly medicine information leaflets [25]. Thus the language of the parental decision aid (v2) was further simplified and visual components were modified to create two engaging and visually appealing resources aimed at a younger audience. For example, color coding was introduced throughout and instructions were added to make the material easier to follow. The original draft version of the decision aid for parents (v2), alongside the drafts made for children and adolescents, were sent to a design team at the university to produce three professionally-branded decision aids.

### 2.3. Validation of the Branded Decision Aids

A mixed panel consisting of pharmacists and parents was recruited through existing contacts to take part in an additional detailed CV exercise (n = 9). Each panel member was interviewed separately by authors GR and OD, who worked through each of the sections of the three branded decision aids seeking qualitative and quantitative feedback on the visual presentation and clarity of the material, and, for the latter two sections of the tools (3: Your feelings, and 4: Your decision) whether these were engaging. Thus, as well as noting specific qualitative comments about each section, the section-specific CVI (S-CVI) for each decision aid was also measured using a four-point response scale as described above.

## 3. Results

The original decision aid aimed at parents (v1) was judged overall to be relevant by the CAMH specialists with a score of 0.96 (>0.80) with 17 of the items deemed to be relevant (I-CVI = 1) before amendments. See Table 1.

The panel recommended rephrasing two items (items 5 and 9) for greater clarity and removing item 12 as it was covered by items 10 and 11. The wording of the decision aid was thus amended in discussion with a CAMH consultant collaborator, to produce the second version of the parental decision aid (v2).

For each of the four sections of the three formatted decision aids, the raters’ assessment of the overall visual presentation and clarity of the material, and, for the latter two sections, whether these were engaging, are shown in Table 2.

A range of qualitative comments and recommendations were made by the panel relating to the appearance of the material (see Table 3), which were taken into account to produce the final versions of all three decision aids.

The final parent, adolescent and child decision aids are shown in the appendices. The parent decision aid (Figure S1) was designed as an A5 booklet for Section 1 and Section 2, with an A4 pull out questionnaire (Figure S2) for self-completion of Section 3 and Section 4. The adolescent decision aid (Figure S3) was designed as a A4 booklet for Section 1 and Section 2, with an A4 pull out questionnaire (Figure S4) for self-completion of Section 3 and Section 4. The child decision aid (Figure S5) was designed as a A4 booklet for reading and self-completion throughout.

## 4. Discussion

This was a unique study that used a sequential method to produce decision aids for use by parents, adolescents, and younger children to decide whether or not a planned drug holiday from methylphenidate is appropriate for their situation. The initial scientific validation of the decision aid with a panel of specialists allowed the team to design and validate three visually appealing and practical documents for use by the different audiences. Interviews with pharmacists and parents allowed detailed feedback to be obtained about the visual presentation, clarity of content, as well as how engaging each component was perceived to be; to enable specific revisions and improvement to be made to further versions. The final version of the parental decision aid was designed as an A5 booklet and pullout self-assessment questionnaire, the adolescent version is similar but with an A4 booklet, and the child version is one A4 booklet for reading and self-completion.

The authors envisage that these materials will open up space for the discussion of drug holidays in ADHD during annual reviews in line with the NICE guidelines [9]. Specifically, we see the material encouraging people to discuss preferences to stop medication, to be involved in any decisions about stopping medication, and to consider trial periods of stopping medication or reducing the dose in cases where the assessment of the overall balance of benefits and harms suggest that this could be appropriate. The authors note that the NICE guidelines now recommend that in fact if a decision is made in a consultation to continue the medication, the reasons for this should be documented [9]. This arguably brings home the absolute importance of the NICE recommendation to review medication and discuss (dis)continuation in a comprehensive assessment with the family. This is not surprising, since methylphenidate has a host of very common and common adverse effects which are not insubstantial in their nature [4]. In fact, the manufacturer warns against prolonged (more than 12 months) and indefinite use of the medication in children and adolescents with a requirement in these cases to monitor the patient for cardiovascular status, growth, appetite, or a worsening of pre-existing psychiatric disorders including (but not limited to) motor or vocal tics, aggressive or hostile behaviour, agitation, anxiety, depression, psychosis, mania, delusions, irritability, lack of spontaneity, withdrawal and excessive perseveration. In addition, if methylphenidate is used for extended periods (over 12 months), the manufacturer also calls for drug holidays at least once yearly [4].

The strengths of this study are that the sequential approach allowed the design of validated and user-ready decision aids. It has to be acknowledged that this study’s limitations are, firstly, that it does not provide any data yet on the practical usefulness and efficacy of the decision aids. Furthermore, another important limitation is the relatively low number of participants who contributed to the validation of the tool. However, in publishing this material, the authors have made available a set of decision aids hitherto missing from the literature and other health resources, which should help health professionals and families to consider and implement drug holidays in ADHD. Future research should look to assess the practical usability of the decision aids in a clinical setting, obtain patient feedback, assess the impact of using these material on decision making processes, the dynamics of use within the clinic setting, as well as ultimately decisions to trial drug holidays and their clinical benefit and quality of life improvements.

## Figures and Tables

**Table 1:** Panel Response for the Item-Level Content Validity Index (I-CVI) of the Parental Decision Aid (v1).

Decision Aid (V1) Item	Item Description (Summarized for the Purpose of This Table)	Relevance of Item (I-CVI)
Section 1**Frequently asked questions**, to provide comprehensive information about planned drug holidays from methylphenidate		
1	The aim of the guide	1
2	Definition of the term “planned drug holiday”	1
3	Reasons for taking a planned drug holiday	1
4	Possible advantages of planned drug holidays	1
5	Possible risks of stopping ADHD medication during drug holidays	0.75 ^a^
6	Best time to consider planned drug holidays from ADHD medication	1
7	Guideline recommendations about drug holidays in ADHD	1
8	What to expect when taking a planned drug holiday	1
Section 2**Compare options**, to explain the risks and benefits of considering a planned break from the medication		
9	Comparing the options	0.75 ^a^
Section 3**Your feelings**, to allow the individual to test their subjective feelings and weigh up their receptiveness to the idea of a drug holiday		
10	My child still needs/does not need the medication (assessing feelings)	1
11	My child may/may not manage well without the medication	1
12	My child has been free of symptoms/still exhibits symptoms	0.75 ^a^
13	I am worried about the possible side-effects/I feel the medication is safe	1
14	I am (not) worried about stopping medication for a short period	1
15	My child is/is not getting appropriate support at school	1
16	I am (not) confident in dealing with my child’s behaviour	1
17	My child is attending college and may (not) need the medication	1
18	We have tried alternative interventions which are (not) effective	1
19	My other important reasons	1
Section 4**Your decision**, to document a final decision about whether to introduce a planned drug holiday from ADHD medication.		
20	The final decision	1

**Notes:** For each item, a question asked the experts to score the relevance of the material on a four-point response scale, providing additional comments where appropriate; with 4 being used to indicate item being very relevant, 3, mostly relevant but needs some revision, 2, mostly not relevant, and needs more revision, and 1, not relevant. The I-CVI was determined by calculating the proportion of panel members who gave a rating of 3 or 4 (relevant or mostly relevant) for each item. Items marked ‘^a^’ did not at first meet minimum acceptable value for I-CVI (<0.80), meaning at least one person of four judged the item to be mostly not relevant or not relevant at all.

**Table 2:** Panel Response for the Section-Level Content Validity Index (S-CVI) of the Branded Decision Aids.

Section of the Decision Aid	Assessment Category	Parent Version S-CVI	Adolescent Version S-CVI	Child Version S-CVI
1 Frequently asked questions	Visual presentation	1.00	0.78 ^a^	1.00
	Clarity of content	0.89	0.56 ^a^	0.89
2 Compare options	Visual presentation	0.89	0.89	0.56 ^a^
	Clarity of content	1.00	0.78	0.56 ^a^
3 Your feelings	Visual presentation	1.00	0.89	0.89
	Clarity of content	0.89	0.67 ^a^	0.56 ^a^
	How engaging	0.78 ^a^	0.67 ^a^	0.67 ^a^
4 Your decision	Visual presentation	1.00	0.78 ^a^	0.89
	Clarity of content	0.78 ^a^	0.56 ^a^	0.89
	How engaging	0.78 ^a^	0.67 ^a^	0.89

**Notes:** For each section, the interviewee recorded a response to “this section is visually appealing” or “this section is clear” or (where relevant) “this section is engaging” on a four-point Likert response scale from (1 = strongly disagree to 4 = strongly agree). The S-CVI was determined by calculating the proportion of interviewees who gave a rating of 3 or 4 (agree/strongly agree) to each of these CV questions. Items marked ‘^a^’ did not at first meet minimum acceptable value for I-CVI (<0.80), meaning more than one person from nine judged the item to be mostly not relevant or not relevant at all.

**Table 3:** Panel Recommendations Implemented to Produce the Final Decision Aids.

Parent Version	Adolescent Version	Child Version
Enlarge font size Reformat some paragraphs Remove bold from some text and embolden other text Improve lay language and presentation Balance volume of text in tables in Section 2 Improve instruction wording Reduce number of examples to 1 in Section 3 Add a double-sided arrow to Section 3 decision statements Add a reference to NICE guidelines in Section 1 Add a sentence to invite a discussion with the clinician at the end of the decision aid	Improve presentation of information boxes in Section 1 Change wording from ‘drug’ to ‘medicine’ Improve instruction wording Balance volume of text in tables in Section 2	Improve title of the decision aid Improve images used to represent adults Change wording for clarity Change visuals to create a simple rating scale for Section 2

## Supplementary Materials

The following are available online at http://www.mdpi.com/2226-4787/6/4/122/s1, Figure S1: A Parent’s Guide: An A5 decision aid booklet; Figure S2: Parents’ guide for decision-making: An A4 pull out questionnaire for self-completion by a parent; Figure S3: Taking a planned holiday from ADHD medicine: An A4 decision aid booklet for adolescents; Figure S4: Helping you decide: An A4 pull out questionnaire for self-completion by an adolescent; Figure S5: Can I take a break from my medication: An A4 booklet for reading and self-completion by a child.
