# Supplementary material for: The Construction and Validation of Child, Adolescent and Parental Decision Aids for Considering Methylphenidate Drug Holidays in ADHD
Source: Pharmacy (Basel). 2018 Nov 24;6(4):122. doi: 10.3390/pharmacy6040122 (PMC6306803; doi:10.3390/pharmacy6040122)
Supplement: Supplementary file 1 [file pharmacy-06-00122-s001.zip › B15172 A5 leaflet Pharmacy ADHD medication decision tool REW 6.pdf]

School of Pharmacy

University of Reading

A PARENTS' GUIDE

Deciding whether or not to consider a planned drug holiday from ADHD medication

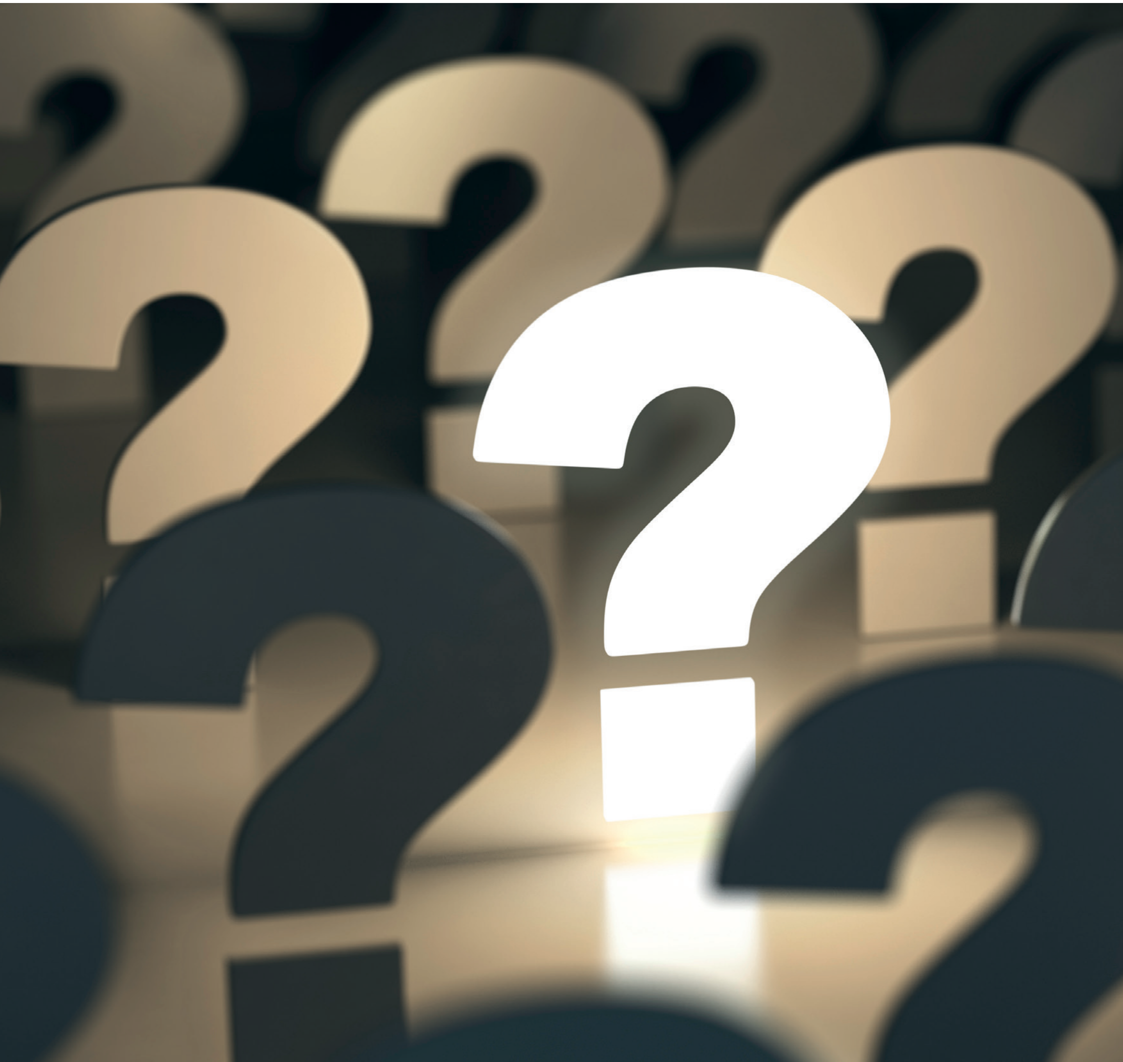

The aim of this guide

This guide aims to provide you with information that could help you decide whether or not you and your child want to introduce planned drug holidays from ADHD medication.

Use this guide in discussions with your child's clinician.

Frequently asked questions (FAQs)

What is the term 'planned drug holiday' referring to?

- **Planned drug holidays** refer to situations where patients 'intentionally stop taking their medication for a defined period of time and for a specific reason'.
- If you were to stop giving your child their ADHD medication for a few days or months on purpose, this would count as a planned drug holiday.
- Ideally, you and your child's clinician need to work together to plan a drug holiday so that you can both assess its benefits.

What are the reasons for taking a planned drug holiday?

- The main reason of planned drug holidays is to **check whether your child still needs the medication** and whether he/she can actually manage without it. A large study about the management of children with ADHD has shown that ADHD medication is less useful after 2-3 years of use. Also, ADHD is a condition associated with children's development and sometimes the hyperactive behaviour of young children can improve as they grow older. For example, around 1 out of 3 children who used to have ADHD will no longer have ADHD symptoms when they become teenagers.
- **Manage and reduce the side-effects of medication** if your child suffers from them. ADHD medication may cause side-effects such as headaches, lack of appetite, sleep difficulties, and slowing the child's growth. Planning breaks from medication (planned drug holidays) could be helpful in easing the medication side-effects – see below.

What are the possible advantages of planned drug holidays?

- The primary advantage is **to avoid continuing with medication if no longer necessary**. A planned drug holiday could be a good opportunity to check if the medication is still needed and whether you and your child can cope well without the medication.
- **Manage the medication side-effects on sleeping and eating.** There is some evidence that stopping methylphenidate at weekends could reduce the undesirable effect of medication on sleeping and eating without increasing child hyperactivity.
- **Allow growth to catch up.** Some research has shown that children who stopped taking ADHD medication during long summer holidays grew much better than those who kept taking the medication.
- **Put your child on a lower dose of medication.** Some children with ADHD need to increase their dose of medication with time. Studies have also shown that stopping the medication temporarily for days, weeks, or months could help place the child back on a lower dose.

What are the possible risks of stopping the ADHD medication during drug holidays?

- By stopping medication, the behaviour and attention deficits may get worse in a child with ADHD. However, there is **no clear evidence about any risks associated with planned drug holidays** from ADHD medication.
- **Planned drug holidays for a short period (e.g. days) do not increase the hyperactive and impulsive behaviour** of children with ADHD. The effects of stopping ADHD medication for longer periods such as during summer holidays and other school holidays are not studied as yet.

What is the best time to consider planned drug holidays from ADHD medication?

- **Summer holidays** or other **school holidays** could be the best time to consider planned drug holidays to avoid causing any difficulties at school.
- However, it could be helpful sometimes to consider planned drug holidays during school days to engage teachers in checking whether your child can cope well at school without the medication.

What are the guideline recommendations in relation to planned drug holidays from ADHD medication?

- The National Institute for Health and Care Excellence (NICE) guidelines advise clinicians to **check regularly, often every year, the continued response to medication** and to plan drug holidays to see if children still need ADHD medication.
- NICE guidelines also advise clinicians to consider planned drug holidays from ADHD medication if child growth has been affected negatively as a result of medication taking.

For more information about the NICE guidelines, visit https://www.nice.org.uk/guidance/cg72/chapter/recommendations

What can you expect if you agree to consider planned drug holidays?

Your child will stop temporarily taking the medication for the period of time that has been agreed through discussion with your child's clinician. This could last between one to two weeks over a school holiday such as at half-term or during the summer holiday.

- **You'll need to watch your child** during the planned drug holiday because you should ideally report your observations back to the clinician.
- **You might notice no difference** in your child's behaviour and attention during the drug holiday and may find that both you and your child can cope well without the medication. If this is the case, then you can discuss with your child's clinician the possibility of stopping medication during school days to also check how your child manages school without medication.
- **Or, you might notice that your child's behaviour is changing** for the worse and that he/she can't cope without the medication. You need to contact your child's clinician in that case, who may advise you to put your child back on medication.

Compare options:

	**Taking** a planned drug holiday from ADHD medication	**Not taking** a planned drug holiday from ADHD medication
**What is usually involved?**	Your child will stop taking his/her medication during a school holiday for a defined period of time which should be agreed with your child's clinician (the break would be for at least 1 or 2 weeks)	Your child will keep taking his/her medication as normally prescribed by your child's doctors.
**What are the benefits?**	Planned drug holidays could be beneficial because they:<br>• Could provide you with actual information about whether your child still needs the medication or not by comparing his/her behaviour and functioning before and after applying the drug holiday.<br>• Could let you see how you and your child may manage without the medication.<br>• Help you avoid continuing with unnecessary medication if it's no longer needed.<br>• Could reduce and manage the medication side-effects.<br>• Could help you put your child on a lower dose of medication.	By continuing to give your child the medication without considering a planned drug holiday, the benefits obtained from medication would continue. For example:<br>• The child will concentrate better<br>• The child's current behaviour will remain more predictable<br>• It will prevent impulsive behaviour
**What are the risks?**	There is no clear evidence in research about the risk associated with planned drug holidays from ADHD medication.<br>However, by not giving children their medication, the hyperactive behaviour and/or attention deficits may return.	Keeping your child on the medication and not taking a planned drug holiday<br>could be linked with risks relating to continued exposure to medication, such as:<br>• The unknown long-term side-effects of ADHD medication.<br>• The short-term side-effects such as: losing appetite, headaches, nausea, sleep problems, slowed growth, heart and mental health problems.<br>• The possibility of keeping your child on unnecessary medication when your child may no longer need the medication.

A PARENTS' GUIDE TO DECISION-MAKING

For more information,please contact:

Dr Parastou Donyai, Division of Pharmacy Practice

University of Reading
Whiteknights
Reading, RG6 6AP

p.donyai@reading.ac.uk
Tel (0118) 378 4704

/theuniversityofreading
@UniofReading

www.reading.ac.uk

Athena SWAN Bronze Award

B15172 02.16
