# Supplementary material for: The Construction and Validation of Child, Adolescent and Parental Decision Aids for Considering Methylphenidate Drug Holidays in ADHD
Source: Pharmacy (Basel). 2018 Nov 24;6(4):122. doi: 10.3390/pharmacy6040122 (PMC6306803; doi:10.3390/pharmacy6040122)
Supplement: Supplementary file 1 [file pharmacy-06-00122-s001.zip › B15481 Adolescent A4 Form Decision-aid tool 3.pdf]

School of Pharmacy

University of Reading

# Taking a planned holiday from ADHD medicine
Helping you decide

**Your feelings:** Your personal feelings are just as important as the medical facts. Think about what matter most to you in this decision. Do this by reading the following statements and placing a tick on the sliding scale to show where your feelings lie.

**Example:**

| *Reasons for considering a planned medicine holiday* | *Reasons for not considering a planned medicine holiday* |
|---|---|

✓

Unsure

## Statement 1

| I feel that I do not need the medication anymore | I feel that I still need the medication |
|---|---|

Unsure

## Statement 2

| I feel that I may manage well without the medication | I am worried that maybe I won't be able to manage without the medication |
|---|---|

Unsure

## Statement 3

| I appreciate the benefits of medication but am worried about the possible short and long-term side-effects | I appreciate the benefits of medication and feel the medication is safe |
|---|---|

Unsure

## Statement 4

| I am not worried about stopping the medication for a short period of time | I would worry about stopping the medication for a short period of time |
|---|---|

Unsure

## Statement 5

I may not need the medication anymore because I am getting the appropriate support at school/college | I still need the medication because I am not getting the appropriate support at school/college

Unsure

## Statement 6

I may not need the medication because I have started attending a school/college and I am enjoying it there | I still need the medication because I have started attending a school/college and medication is necessary for improving my concentration

Unsure

## Statement 7

I may not need the medication anymore because I have been using alternative interventions (such as my changing diet) and these have proved to be effective | I still need the medication because I have tried different alternative interventions (such as my changing diet) and nothing has proved to be effective

Unsure

## Other important reasons.

Use this box to write down any other important reasons that matter most to you in this decision:

## Your decision:

Now that you've thought about the facts and your feelings, you may have a general idea of where you stand on this decision of whether to take a planned drug holiday or not. Show which way you are leaning towards right now:

**Agree to try a planned drug holiday from medication** | **Keep on medication and not try a planned drug holiday**

| | | | | | | |
|---|---|---|---|---|---|---|
| | | | | | | |

More important | Equally important | More important

## Speak to your family and doctor about your decision.

## You have now finished using this decision aid, thank you for your time.
