# Supplementary material for: The Construction and Validation of Child, Adolescent and Parental Decision Aids for Considering Methylphenidate Drug Holidays in ADHD
Source: Pharmacy (Basel). 2018 Nov 24;6(4):122. doi: 10.3390/pharmacy6040122 (PMC6306803; doi:10.3390/pharmacy6040122)
Supplement: Supplementary file 1 [file pharmacy-06-00122-s001.zip › B15172 A4 form Pharmacy ADHD medication decision tool REW 3.pdf]

School of Pharmacy

University of Reading

Deciding whether or not to consider a planned drug holiday from ADHD medication: parents' guide for decision-making

Your feelings: Your personal feelings are just as important as the medical facts. Think about what matters most to you in this decision. Do this by reading the following statements and placing a tick on the sliding scale to show where your feelings lie.

Examples:

Reasons for considering a planned drug holiday		*Reasons for not considering a planned drug holiday*
More important	Unsure ✓	More important

Statement 1

**I feel my child does not need the medication anymore**		**I feel my child still needs the medication**
More important	Unsure	More important

Statement 2

**I feel my child and I may manage well without the medication**		**I am worried that my child and maybe I won't be able to manage without the medication**
More important	Unsure	More important

Statement 3

**I appreciate the benefits of medication but am worried about the possible short and long-term side-effects**		**I appreciate the benefits of medication and feel the medication is safe**
More important	Unsure	More important

Statement 4

**I am not worried about stopping the medication for a short period of time**		**I am worried about stopping the medication even for a short period of time**
More important	Unsure	More important

Statement 5

My child may not need the medication anymore because he/she is getting the appropriate support at school | **My child still needs the medication because he/she is not getting the appropriate support at school**

More important — Unsure — More important

Statement 6

My child may not need the medication because I feel confident in dealing with my child's behaviour | **My child still needs the medication because I still find it difficult to deal with my child's behaviour**

More important — Unsure — More important

Statement 7

My child may not need the medication because he/she started attending a college and is enjoying the course | **My child still needs the medication because he/she started attending a college and medication is necessary for improving his/her concentration**

More important — Unsure — More important

Statement 8

May child may not need the medication anymore because we have been using alternative interventions (such as changing diet) and these have proved to be effective | **My child still needs the medication because we have tried different alternative interventions (such as changing diet) and nothing has proved to be effective**

More important — Unsure — More important

Other important reasons.

Use this box to write down any other important reasons that matter most to you in this decision:

Your decision:

Now that you've thought about the facts and your feelings, you may have a general idea of where you stand on this decision. Show which way you are leaning towards right now:

Agree to try a planned drug holiday from my child's medication | **Keep my child on medication and not try a planned drug holiday**

More important — Equally important — More important

Speak to your child's clinician about your decision and feelings.

You have now finished using this decision aid, thank you for your time.

B15172 02.16
