# Supplementary material for: The Construction and Validation of Child, Adolescent and Parental Decision Aids for Considering Methylphenidate Drug Holidays in ADHD
Source: Pharmacy (Basel). 2018 Nov 24;6(4):122. doi: 10.3390/pharmacy6040122 (PMC6306803; doi:10.3390/pharmacy6040122)
Supplement: Supplementary file 1 [file pharmacy-06-00122-s001.zip › B15481 Adolescent A4 Leaflet Decision-aid tool 5.pdf]

School of Pharmacy

University of Reading

TAKING A PLANNED HOLIDAY FROM ADHD MEDICINE

Helping you decide about taking a medicine holiday

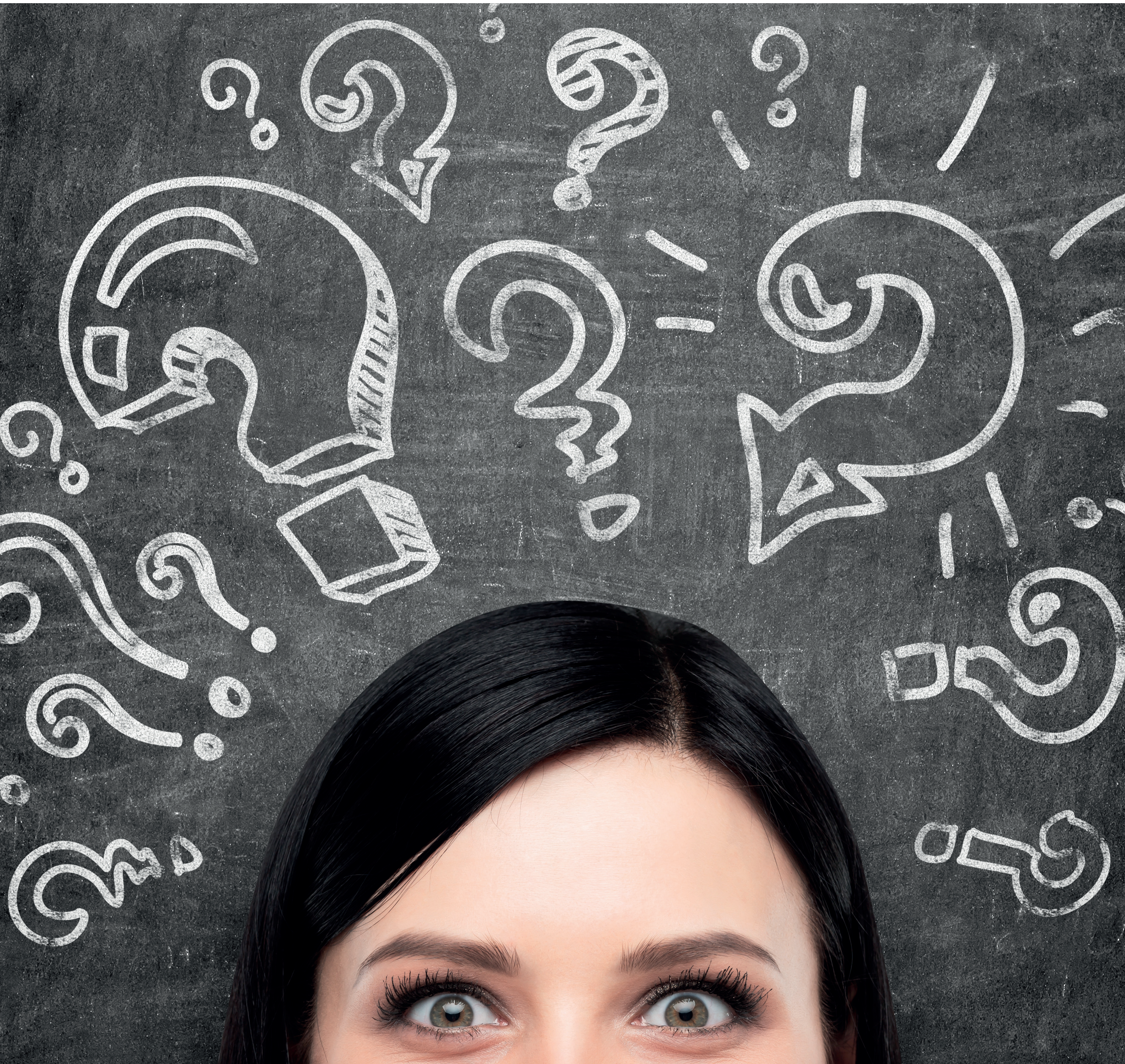

The aim of this guide

You have been identified by your doctor as a patient who is suitable to take a medicine holiday. You may be suitable for a medicine holiday due to the length of time you have been taking medication for your ADHD.

You currently take medication, for your condition ADHD, which helps to improve your behaviour and concentration. This booklet will help you learn about considering a medicine holiday in ADHD.

Ideally, you should read this guide with your parents and discuss your decision with your doctor. Here are some questions to think about to help you understand about medicine holidays.

A medicine holiday is when 'a patient stops taking their medication deliberately for a defined period of time for a specific reason'

What are the reasons for considering a medicine holiday?

The main reason of a planned medicine holiday is to check whether you still need the medication and whether you can actually manage without it.

The medicine holiday may manage and reduce the side-effects of medication if you suffer from them. ADHD medication may cause side-effects such as headaches, lack of appetite, sleep difficulties and slow growth.

The facts about a medicine holiday

The benefits

- It avoids continuing with medication if not longer necessary
- It reduces the medication side effects on sleeping and eating
- You may need to take less medicine or even stop your medicine forever, as may no longer need it after your break. Both of these changes could help improve your growth

The risks

- There is a possibility that by stopping medication, your behaviour and concentration levels may get worse.

When would a medicine holiday be taken?

Summer holidays or other school/college holidays could be the best time to consider planned medicine holidays to avoid causing any difficulties at school or college.

What would I expect if I decided to take a medicine holiday?

- You will stop temporarily taking the medicine.
- You will have to make note of any changes during your medicine holiday – you may notice no difference or that your behaviour is getting worse.
- You will report your experience and feelings to your doctor.

What is usually involved?

Taking a medicine holiday

- You will stop taking your medication during a school holiday for a defined period of time
- You will agree with your family and doctor, a period of time to be off your medication

Not taking a medicine holiday

- You will keep taking your as normally prescribed by your doctor

What are the benefits?

The benefits of taking a medicine holiday

- Could reduce and manage the medication's side-effects
- Could let you see how you may manage without the medication
- Would help you avoid continuing with unnecessary medication if it's no longer needed
- Could help put you on a lower dose of medication

The benefits of not taking a medicine holiday

- You will be taking your medication as normally prescribed by your doctors

What are the risks?

The risks of taking a medicine holiday

- By not taking medication, the hyperactive behaviour and/or attention deficits may return

The risks of not taking a medicine holiday

- The long-term side-effects of the ADHD medication will be unknown
- The short-term side-effects such as: losing appetite, headaches and sleep problems may continue
- You may be staying on unnecessary medication when you may no longer need it

Use the form to help you make your decision

HELPING YOU DECIDE ABOUT TAKING A HOLIDAY FROM ADHD MEDICINE

For more information, please contact:

Dr Parastou Donyai, Division of Pharmacy Practice

University of Reading
Whiteknights
Reading, RG6 6AP

p.donyai@reading.ac.uk
Tel (0118) 378 4704

/theuniversityofreading
@UniofReading

www.reading.ac.uk

Athena SWAN Bronze Award

B15481 02.16
