# Supplementary material for: The Construction and Validation of Child, Adolescent and Parental Decision Aids for Considering Methylphenidate Drug Holidays in ADHD
Source: Pharmacy (Basel). 2018 Nov 24;6(4):122. doi: 10.3390/pharmacy6040122 (PMC6306803; doi:10.3390/pharmacy6040122)
Supplement: Supplementary file 1 [file pharmacy-06-00122-s001.zip › B15481 Child A4 Decision-aid tool 3.pdf]

School of Pharmacy

University of Reading

Can I take a break from my ADHD medicine?

Read this booklet with your parents to find out more!

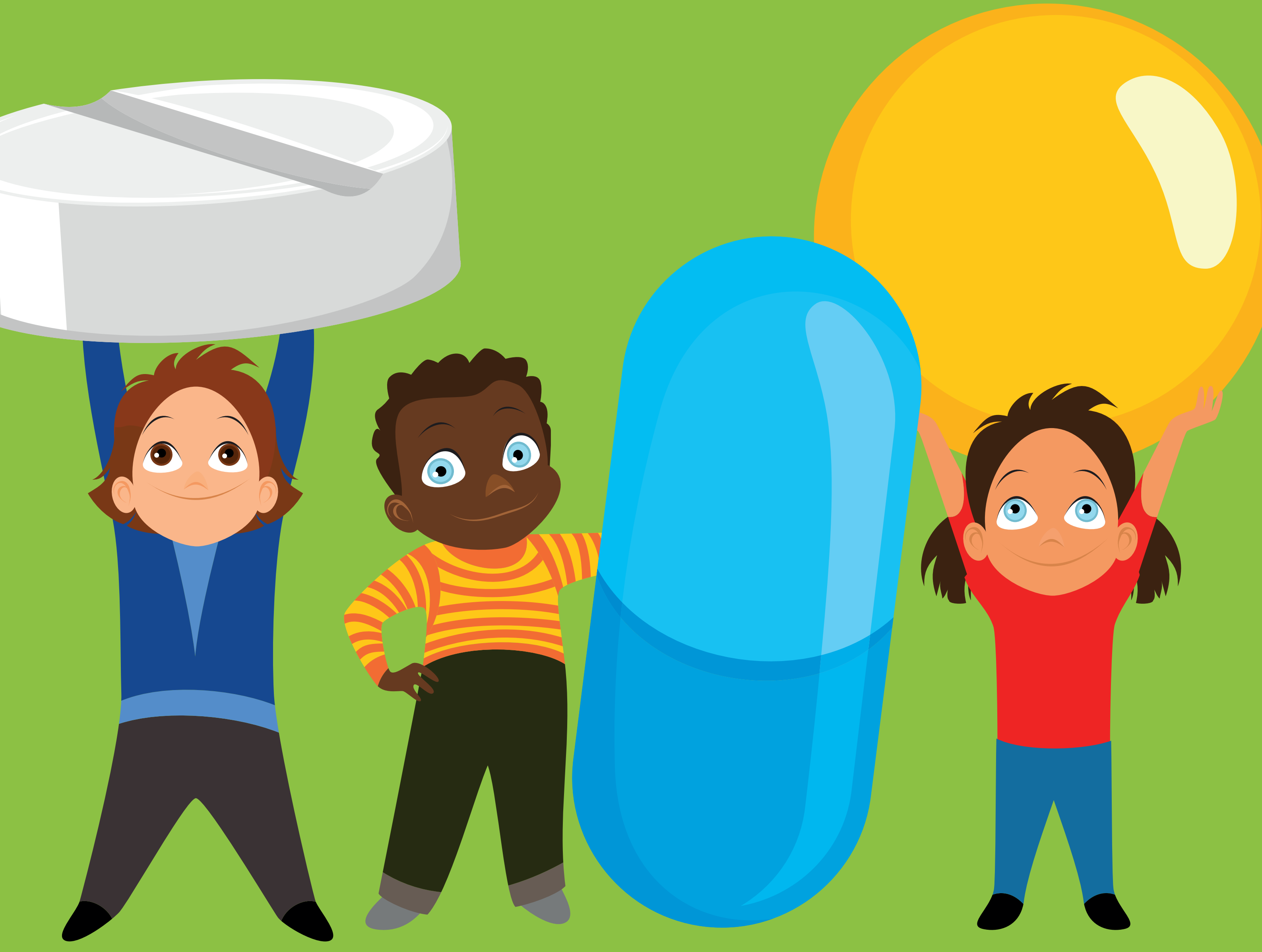

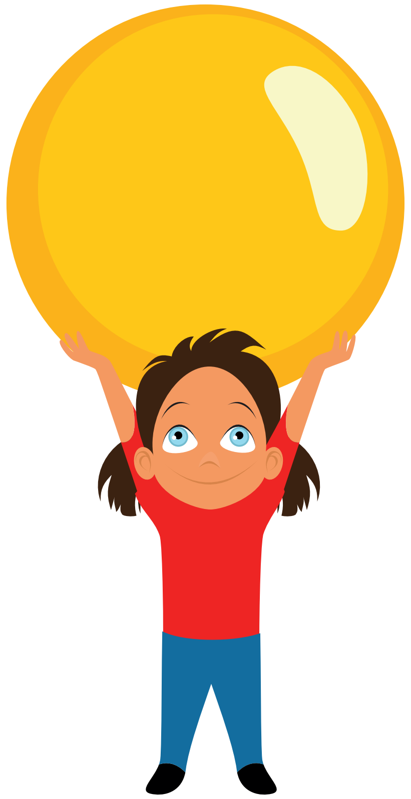

What is this booklet for?

You take medicine for your condition which is called ADHD.

Your medicine helps you to concentrate better.

This booklet is to help you, your family and your doctor to decide about whether or not you should take a break from your ADHD medicine.

Read this booklet with your family.

If there is anything you don't understand or you have any questions, let your family know.

Now it is time to learn about what a medicine holiday is!

Hi there! I am a doctor.
I call a break from medicine a **planned medicine holiday**.
I help people like you to decide if they want to take a break from their medicine.

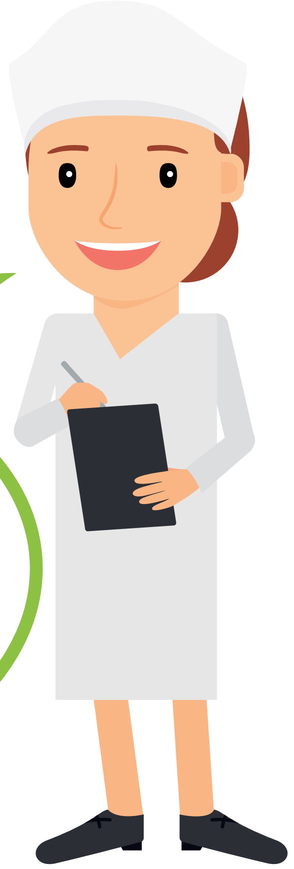

Doctors are here to help you decide about a medicine holiday as sometimes you may have problems with your medicines. You may get headaches or not feel hungry while on your ADHD medicine and therefore a break may stop these problems.

Try this puzzle

Here is a word search for you to try.

It will help you learn words that will be used in this booklet.

D	U	Z	W	S	F	Z	A	J	O
E	S	D	O	C	T	O	R	L	T
C	L	M	E	D	I	C	I	N	E
I	B	U	M	A	K	H	P	C	R
S	J	R	N	K	K	O	L	A	U
I	S	T	E	H	N	L	J	S	X
O	B	E	H	A	V	I	O	U	R
N	R	N	U	D	K	D	T	S	S
B	X	C	V	H	V	A	O	I	W
J	R	Q	K	D	I	Y	F	K	B

ADHD
BEHAVIOUR
BREAK
DECISION
DOCTOR
HOLIDAY
MEDICINE

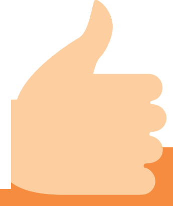

Good things about a medicine holiday

- ✓ The break will help you stop problems caused by your medicine such as finding it hard to sleep or getting headaches.
- ✓ It has been found by scientists that ADHD medicine may reduce how tall you are going to be.
- ✓ You may need to take less medicine or even stop your medicine forever, as may no longer need it after you break. Both of these changes could help improve your growth.

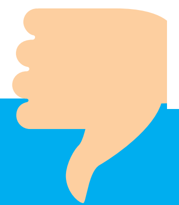

Bad things about a medicine holiday

- ✗ You may find it hard to concentrate at school or home, but this doesn't usually happen in medicine breaks because they are only for a small amount of time.

When is the best time to have my break from medicine?

Summer holidays or school holidays are usually the best time to take a break from your medicine as it won't affect your concentration at school

What will happen if I want to have a break from my medicine?

If you agree to take a break from your medicine, you will stop taking it for a set time → You may not see any difference in the way you act or you may feel different → If you feel different and you don't like it, let your family or doctor know

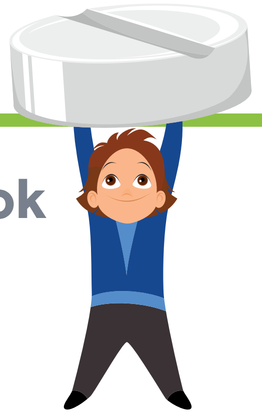

Now you have finished reading this, take a look through some questions on the next page to think about how you feel.

How do you feel?

It is important that you are always happy and make the right decision for you. Read the sentences below and think about how they make you feel.

Instructions

If you agree more with the **blue sentence** you can tick ✓ the box below the **blue smiley face.**

If you agree more with the **purple sentence** you can tick ✓ the box below the **purple smiley face**.

If you are unsure how you feel about the sentence or think they are both as important as each other, you can tick ✓ the box below the **grey smiley face**.

Let's begin!

Reasons for wanting a break from ADHD medicine		Reasons for not wanting a break from ADHD medicine
☺	☺?	☺

Part 1:

I feel I do not need the medicine anymore		I feel I still need the medicine
☐	☐ Unsure	☐

Part 2:

I will be happy without my medicine		I will be unhappy without my medicine
☐	☐ Unsure	☐

Part 3:

I am not worried about stopping the medicine for a short period of time

I am worried about stopping the medicine even for a short period of time

☐ ☐ Unsure ☐

Part 4:

I may not need the medicine anymore because I am getting help at school

I still need the medicine because I am not getting enough help at school

☐ ☐ Unsure ☐

Part 5:

I may not need the medicine because I am getting on well at school and enjoy it

I still need the medicine because I am not doing well at school and can't concentrate without it

☐ ☐ Unsure ☐

Part 6:

My other important reasons for wanting to take **or** not wanting to take a drug holiday:

Your decision:

You have now had time to think about the facts and your feelings.

By now, you may have an idea about if you want to take a break from your medicine or not.

Show which way you are feeling right now with a tick ✓ in the box below:

I would like to try a break from my medicine

I want to stay on my medicine and not have a break

☐ Leaning towards this

☐ Undecided

☐ Leaning towards this

Speak to your family and doctor about which one you picked.

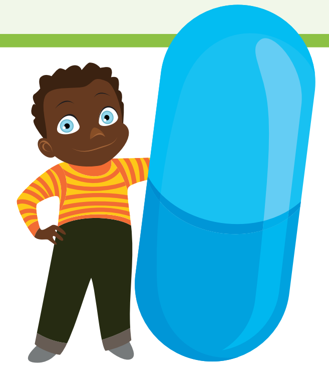

You have now finished reading the booklet. Thank you for taking the time to read it!

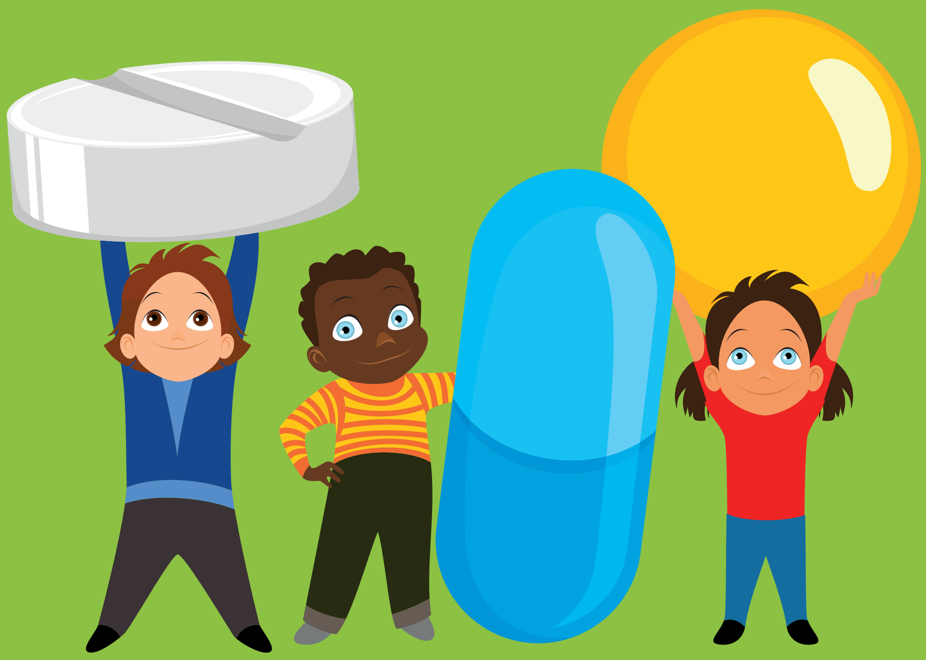

CAN I TAKE A BREAK FROM MY ADHD MEDICINE?

For more information, please contact:

Dr Parastou Donyai, Division of Pharmacy Practice

University of Reading
Whiteknights
Reading, RG6 6AP

p.donyai@reading.ac.uk
Tel (0118) 378 4704

/theuniversityofreading
@UniofReading

www.reading.ac.uk

Athena SWAN Bronze Award

B15481 02.16
